# A Concern for Intraoperative Distractions and Interference: An Observational Study Identifying, Measuring, and Quantifying Both within the Operating Theatre

**DOI:** 10.1155/2021/9910290

**Published:** 2021-12-11

**Authors:** Shane Keogh, Deirdre Laski

**Affiliations:** Department of General Surgery, Our Lady of Lourdes Hospital, Drogheda, Drogheda Co., Louth A92 VW28, UK

## Abstract

**Background:**

Modern surgical research has broadened to include an interest into the investigation of surgical workflow. Rigorous analysis of the surgical process has a particular focus on distractions. Operating theatres are inherently full of distractions, many not pertinent to the surgical process. Distractions have the potential to increase surgeon stress, operative time, and complications. Our study aims to objectively identify, classify, and quantify distractions during the surgical process.

**Methods:**

46 general surgical procedures were observed within a tertiary Irish hospital between June 2019 and October 2019. An established observational tool was used to apply a structured observation to all operations. Additionally, a nine-point ordinal behaviourally anchor scoring scale was used to assign an interference level to each distraction.

**Results:**

The total operative observation time was 4605 minutes (mean = 100.11 minutes, std. deviation: 45.6 minutes). Overall, 855 intraoperative distractions were coded. On average, 18.58 distractions were coded per operation (std. deviation: 6.649; range: 5–34), with 11.14 distractions occurring per hour. Entering/exiting (*n* = 380, 42.88%) and case irrelevant communication (*n* = 251, 28.32%) occurred most frequently. Disruption rate was highest within the first (*n* = 275, 32%) and fourth operative quartiles (*n* = 342, 41%). Highest interference rates were observed from equipment issue and procedural interruptions. Anaesthetists initiated CIC more frequently (2.72 per operation), compared to nurses (1.57) and surgeons (1.17).

**Conclusion:**

Our results confirm that distractions are prevalent within the operating theatre. Distractions contribute to significant interferences of surgical workflow. Steps can be taken to reduce overall prevalence and interference level by drawing upon a systems-based perspective. However, due to the ubiquitous nature of distractions, surgeons may need to develop skills to help them resume interrupted primary tasks so as to negate the effects distraction has on surgical outcomes. Data for the above have been presented as conference abstract in 28th International Congress of the European Association for Endoscopic Surgery (EAES) Virtual Congress, 23–26 June 2020.

## 1. Introduction

Modern day surgery is becoming increasingly complex, requiring higher levels of concentration to compliment the growing surgical skillset. Recently, research has broadened to include an interest into surgical workflow, prompting rigorous analysis of the surgical process, with particular focus on the effects of distractions in operating theatres (OTs).

Modern OTs inherently are full of distractions, many not pertinent to the surgical process [1]. Studies outside of health care in similar high-risk industries (e.g., aviation/nuclear/automotive) have consistently demonstrated that distractions have detrimental consequences on performance and generate a true risk to public safety [2]. Research regarding distractions in the OT is still in the early stages but is starting to be addressed [3–10].

An intraoperative distraction is defined as an event during the surgical process that potentially distracts the surgeon and/or team member from their primary task and subsequently can lead to interruptions of the surgical process [11]. Despite being complex and multifactorial in origin, distractions in the OT can be quantified and qualified. Experimental setting studies have demonstrated the negative effects that distractions have on performance in the OT. Distractions during the surgical process obstruct the safe and timely completion of operations, leading to team inefficiency, increased operative time, and, more worrisome, an increase in operative complications such as visceral injury and haemorrhage [4, 6, 12–14]. More recent studies have also demonstrated the unique effects that distractions have on the operating surgeon, increasing mental fatigue reported per case, increasing situational stress, and reducing overall performance. The recent development of robotic systems introduces a range of new surgical and technical demands, which are also effected negatively by distractions [4].

Thus, it is of paramount importance to explore the occurrence and impact of distraction in real-time surgical procedures. Our study aims to objectively identify, classify, and quantify distractions during the surgical process and to contribute to the growing evidence base regarding the effects of intraoperative interruptions on the surgical process. Specifically, we aim to

1. Identify and classify the types of distractions that occur during surgical procedures
2. Quantify frequency and timing of interruptions to identify trends/phases with increased interruption levels during the surgical process
3. Identify how different operators contribute to case irrelevant communication (CIC)

## 2. Materials and Methods

### 2.1. Design

An established observational tool developed by Sevdalis et al. [5] was used to apply a structured observation to all operations included. Structured observational research involves monitoring of healthcare domains and collection of pertinent data such as errors, near misses, team performance, and organisational culture with the aim of identifying individual, team, and organisational precursors for adverse events. IRB approval was not needed, and patient and staff consent was not sought as identities and personal details were not recorded during the observations.

We defined distraction as the observed event, which caused the surgical team to orientate away from a primary task and an interruption as a distraction resulting in a break in primary task activity. The disruptions observed in the OT were captured with a predefined coding scheme dividing interruptions into seven predefined sources: (1) people entering/exiting the OT, (2) phone/bleep calls, (3) case irrelevant communication (CIC), (4) equipment issues/failure, (5) work environment/ergonomic (e.g., laparoscopic screen placed in wrong place), (6) movement (e.g., around patient to fix ECG leads), and (7) procedural factors. A nine-point ordinal behaviourally anchor scoring scale, previously described by Healey et al., was used to assign an interference level to each distracting event based on the extent the disruption interrupted the OT team and overall OT functioning [15], highlighted in Table 1. A score of 1 represents a potentially distracting source not followed by a response, and the highest score, 9, represents a stimulus that causes complete interruption of the primary task and operative flow disruption. Additionally, observers noted the following information: time of disruptive events relative to start time and surgical team members initiating the disruption.

### 2.2. Study Setting and Sample

Surgical cases were observed at an established Irish University Hospital as an internal review on OT environment from June 2019 to October 2019. The study included both “elective” and “emergency out of hours” procedures performed within a general surgical department in order to broaden the data. The observed intraoperative time, from “surgical sign in” to “sign out” as per the WHO guidelines, was noted. Two OTs were used during the study, and team composition varied. However, there was generally consistency in surgical and nursing team members, as personnel were assigned to particular OTs within the hospital. The OT team was identified as the staff assigned to individual observed surgical case and comprised three main professions; (1) anaesthetists and their assistants, including an anaesthesia nurse; (2) an assigned nursing group consisting of a sterile scrubbed nurse and circulating nurse; and (3) the surgeons, including the operating and assisting surgeon. Furthermore, all OTs and observed cases were comparable in terms of size, equipment available, positioning of equipment, and staffing levels.

### 2.3. Data Collection Procedure and Analysis

Two data collectors, authors SK and DL, were trained prior to the study. SK and DL jointly conducted six pilot observations. Both observers presented to the OT prior to the operation and stayed for the duration of the procedure, positioning themselves, so that the whole OT and all OT team members could be observed. The observer did not interact with OT staff and did not cause any recordable interruptive events. All observations were recorded manually on adapted observation sheets. Observed data collected were double-entered into a database. All statistical calculations were performed using JASP software (version 0.11.1, University of Amsterdam, Netherlands). For all analyses, a *P* value < 0.05 was considered statistically significant.

## 3. Results

A total of 46 surgical procedures were observed within the study period, limited by time and staffing constraints. These procedures consisted of “elective” operative cases (*n* = 20) and emergency “out of hours” cases (*n* = 27). They were chosen primarily based on availability of staff to conduct the observation. The total operative observation time was 4605 minutes (76.75 hours), with a mean operative case duration of 100.11 minutes and operative time range of 20–288 minutes. Seven operation types were observed: laparoscopic appendectomy, laparoscopic cholecystectomy, incision and drainage of abscess, laparoscopic unilateral inguinal hernia repair, open unilateral inguinal hernia repair, reversal of Hartman's, and emergency laparotomy.

### 3.1. Sources of Intraoperative Surgical Flow Disruptions

Overall, 855 intraoperative distractions were coded within the observed operations. On average, 18.58 distractions were coded per surgical procedure (std. deviation: 6.649; range: 5–34). With a total of 855 intraoperative operations being coded over a total of 4605 minutes (76.75 hours), an average of 11.14 distractions occurred per hour within the operative time observed. Of all the observed distraction groups coded, people entering/exiting the room (*n* = 380, 42.88%) and CIC (*n* = 251, 28.32%) accounted for the highest absolute number of observed distractions. Other distractions included phone/bleep distractions (*n* = 72, 8.12%), equipment issue/failure (*n* = 32, 3.61%), work environment/ergonomic distractions (*n* = 32, 3.61%), movement distractions (*n* = 89, 10.04%), and procedural distractions (*n* = 30, 3.38%).  Table 2 presents the prevalence of distractions per hour, with people entering the OT being the most prevalent one occurring on average 5.13 times per hour.

**Figure 1:**
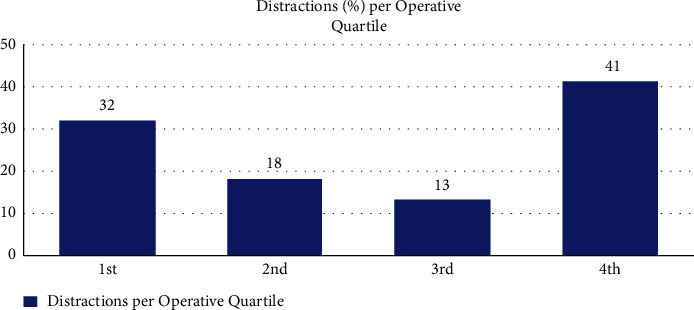
Timed distractions during the course of 46 surgical procedures divided into quartiles (*n* = % of total distractions observed).

Additionally reported in Table 2 are the average distraction levels coded per group. High interference rates were observed from the distractions triggered by equipment issue/failure (average interference rate per event 6.8, std. deviation: 1.23) and procedural interruptions (average interference rate per event 6.5, std. deviation: 1.24). Comparatively low interference rates were observed with movement in the OT and phone/bleep distractions. 3009 interference points were recorded with a mean interference rate per case being 65.41 (range: 10–121, std. deviation: 27.311).  Figure 1 compares the total distraction frequency per source to the average interference per source.

### 3.2. Intraoperative Disruptions and Time Variation

**Figure 2:**
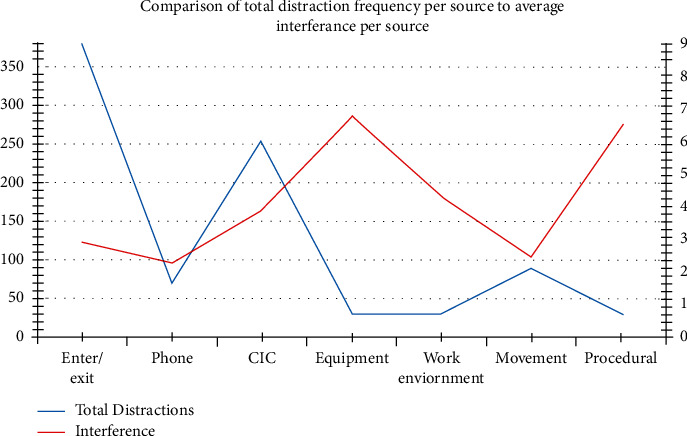
Total observed distractions per source (*n* = total number) vs. average interference per source of distraction (scale 1–9).

We additionally coded the time at which disruptions occurred during the operative time, time zero indicating “knife to skin.” As with other research studies of this kind, operative time was divided into quartiles to further assess the time periods at which disruptions occurred. The frequency of disruptions was highest within the first (*n* = 275, 32%) and fourth quartiles (*n* = 342, 41%) (Figure 2). The total cumulative interference levels similarly were higher in the first (*n* = 849) and fourth quartiles (*n* = 1068), respectively. Despite cumulatively higher levels in the first and fourth quartile, events that produced the highest distraction levels, equipment issue/failure, and procedural issues occurred more frequently in the second (40.62%) and third quartile (53.12%). Additionally, there was a positive correlation between total time of the operation and the total number of distractions observed (Pearson's correlation (*r*) = 0.515, *P* < 0.001) and the total distraction level per case (*r* = 0.569, *P* < 0.001).

### 3.3. Intraoperative CIC Relationships to OT Professionals

CIC (case irrelevant communication) occurred for a total of 251 times within the observed operating times, at an average of 3.27 times per hour and 5.52 times per operation (range: 2–13). We additionally coded the disruptions with regard to the health care professional who initiated the CIC (anaesthetist, nurse, and surgeon). With regard to CIC interruptions, the observation show that anaesthetists initiate the CIC more frequently with 2.72 CIC per operation (range: 0–7, std. deviation: 1.85), compared to nurses 1.57 (range: 0–8, std. deviation: 1.67) and surgeons 1.17 (range: 0–6, std. deviation: 1.43).

## 4. Discussion

By applying a structured observation within this study, we aimed to investigate the character, prevalence, and interruption level of distractions within a general surgical OT. By exploring data from 46 operative procedures over a total cumulative time period of 76.75 hours, we demonstrated that within the OT, there is a high prevalence of distracting events, amounting to a high level of interruptions on surgical workflow. Our results confirm that distractions are inherent within the OT environment as seen with previous studies [1, 2, 5, 15, 16].

To contribute to the growing evidence that distractions within the OT interfere with effective surgical practice and workflow, we aimed to assess the source of distractions to allow a better understanding of their origins within the OT. On average, 11.14 distractions occurred per hour in the operating environment and intraoperative interruptions most frequently occurred as a result of people entering and exiting to OT and CIC. This relative contribution per observed source mirrors the previous published literature [2, 5, 15, 16]. CIC occurred 251 times within 42 cases at a rate of 3.27 events per hour, and similarly, Healy et al. and Sevdalis et al. demonstrated the high frequency of CIC occurring in OT [1, 15]. This supports claims that there are high levels of irrelevant communication in OT. Our study provides insight into the working of a surgical team as a functional unit by addressing the origins of CIC. Surgeons have previously been shown to play a central part in CIC and have been identified as the key initiators of CIC [5]. Unlike previous studies, we identified that anaesthetists and nurses initiate CIC at higher frequencies in the OT. Surgeons initiated the least interruptions attributed to CIC. CIC has been shown to be detrimental to surgical workflow, and surgeon performance is hindered by CIC initiated by other healthcare professionals in the OT [3, 7, 12, 17]. Healy et al. identified that surgeons recognise the compromised performance that CIC causes, and CIC initiated by other healthcare professional can perpetuate conflict within the OT [5]. Conflict within the OT work environment can further lead to increased stress, reduced collaboration, and failure of surgical workflow.

The concept of a sterile cockpit has been successful in reducing CIC within the aviation industry and has been proposed to be introduced within the OT. Nevertheless, in spite of its success in similar industries, CIC within the OT may attribute positively to outcomes. CIC may mitigate the escalation of stress within surgeries while at the same time improving team task outcomes, increasing rapport, and creating an overarching positive social atmosphere within the OT [5, 13]. Therefore further research is needed to address the boundaries needed on restrictive measures to prevent CIC distractions within the OT.

The prevalence of observed distraction events forms a system analysis perspective but does not highlight the effect of distractions on the OT team. The observation of interference level highlights how distractions interfere with a functioning OT. Overall, interference levels per procedure were high and comparable to previous studies [2, 15, 18]. We additionally have shown that high distraction prevalence did not correlate with high interference levels, with the highest disruption level per distraction, equipment failure, occurring infrequently. This is supported by previous findings identifying that equipment issues and procedural distractions disrupt surgical workflow and outcomes more severely but are less frequent in their occurrence. Distractions from the above disrupt at a higher level, as they require the whole surgical team to redirect their collective attention from the surgery, the primary task, to a secondary task. Psychological research has shown that switching tasks from a primary to secondary task reduces outcomes of the primary task, thus highlighting the importance of maintaining a continuity of concentration [8, 16, 19]. Surgery is constantly developing with ever-expanding technology requiring surgeons to learn and re-learn different skill sets, making them vulnerable to distraction. Recent studies show that robotic surgery is also victim to distractions. Preoperative equipment testing to mitigate these high interference distractions is important to maintain efficient surgical workflow. People entering and exiting contributed to the highest number of distributions observed but low absolute levels of distractions per event. The high level of entering and exiting was exacerbated when equipment was not on hand to the scrubbed nurse. We observed that multiple interruptions originated from the retrieval of equipment not stored in the OT. The time at which the OT door was open directly contributed to increased environmental distraction, and despite these distractions not causing disruptions of a high level, their frequency can be reduced with improved resource planning. The above two observations call for careful purposeful preoperative preparation to reduce overall and absolute distractions that occur in the OT.

Distractions have previously been shown to occur more frequently within the first half of operations. We identified an initial peak in distractions within the first quartile of the procedure, followed by a nadir in the second and third quartiles and a subsequent second peak within the fourth quartile. This pattern of distractions can have specific negative effects on surgical outcomes. The initial peak in distractions is of concern as higher initial frequency of distractions has been linked to higher overall stress levels in surgeons [11]. Reduced surgical performance is likely as initially strategic planning, critical decision-making, and resource planning will be interrupted within the first quartile and subsequently patient postoperative planning will be disrupted in the final quartile. If performance in these critical times is hindered by distractions and interruptions, patient safety and outcomes will be compromised.

Overall, our results highlight that distractions are prevalent in the modern OT. These distractions contribute to significant interferences of surgical workflow. Consequently, interferences within complex surgical procedures may affect surgeon performance and negatively affect surgical patient-orientated outcomes. Due to the ubiquitous nature of distractions, surgeons may need to develop new and unique skills to help them resume interrupted primary tasks so to negate the effects distractions have on surgical outcomes. However, it should not be expected for surgeons to deal with increasing distractible work conditions, as there is a limit to what individuals may adapt to. Ultimately, a broader awareness of distraction and interruption within OT may guide the improvement of surgical processes by reducing overall prevalence of disruptions and interference level by drawing upon a systems-based perspective to look at surgery as a whole, from start to finish and team to patient.

## Figures and Tables

**Table 1:** Ordinal scale used to assign an interference score to each observed distraction.

Interference level	Observable effect on team member or team functioning
1.	Potentially distracting sources (e.g., beeper call but no one responds to it)
2.	Interference noticed by floating personnel (e.g., beeper call is noticed by the circulating nurses but not dealt with)
3.	Floating member attends to noncase distraction (e.g., the circulating nurse responds to the beeper call)
4.	Single team member momentarily distracted from the task (e.g., anaesthesiologist orients away from the focal tasks of documentation to a beeper call while continuing with the documentation)
5.	Team member pauses current task
6.	Team member attends to distraction (e.g., surgeon responds to queries about the next case)
7.	Team distracted momentarily
8.	Team attends to distraction
9.	Operation flow disrupted (e.g., equipment failure that stops the surgical procedure)

**Table 2:** Observed intraoperative distraction (total and frequency) and interference per disruption source.

Intraoperative disruption source		Observed intraoperative distractions					Interference	
		*n*	%	Total cases	Max per case	Per hour	Average interference, 1–9 (std. deviation)	Total interference
1.	People entering/exiting	380	42.8	46	18	4.95	2.9 (1.34)	1102
2.	Phone/bleep	72	8.1	34	5	0.93	2.3 (1.62)	165
3.	Case irrelevant communication (CIC)	251	28.3	42	13	3.27	3.9 (1.41)	979
4.	Equipment issues/failure	32	3.6	7	3	0.41	6.8 (1.23)	218
5.	Work environment/ergonomic	32	3.6	16	2	0.41	4.3 (2.44)	137
6.	Movement	89	10	33	6	1.15	2.4 (1.32)	213
7.	Procedural interruption	10	3.3	12	4	0.39	6.5 (1.24)	195

## Data Availability

All the data underlying the findings are fully available without restrictions.
